# Influence of acclimation to sublethal temperature on heat tolerance of *Tribolium castaneum* (Herbst) (Coleoptera: Tenebrionidae) exposed to 50°C

**DOI:** 10.1371/journal.pone.0182269

**Published:** 2017-08-07

**Authors:** Jianhua Lü, Shuli Liu

**Affiliations:** Province Key Laboratory of Transformation and Utilization of Cereal Resource, School of Food Science and Technology, Henan University of Technology, Zhengzhou High-Tech Development Zone, Zhengzhou, Henan, China; Chinese Academy of Sciences, CHINA

## Abstract

*Tribolium castaneum* (Herbst) (Coleoptera: Tenebrionidae) is a serious pest of stored agricultural products and one of the most common insects found in grain storage and food processing facilities. Heat treatment has been revisited to control stored-product insects as a potential alternative to methyl bromide for disinfesting mills and food-processing facilities. The influence of acclimation of *T*. *castaneum* adults, pupae, larvae, and eggs to sublethal temperatures of 36, and 42°C on their subsequent susceptibility to lethal temperature of 50°C was respectively investigated. The acclimation of *T*. *castaneum* eggs, larvae, pupae, and adults to 36, and 42°C significantly decreased their subsequent susceptibility to lethal high temperature of 50°C. The influence of acclimation to 42°C was significantly greater than that of acclimation to 36°C. The most influential acclimation times at 42°C for mortality of *T*. *castaneum* eggs, larvae, pupae, and adults were 15, 5, 5, and 5 h, respectively, and their corresponding mortality were 41.24, 5.59, 20.19, and 4.48%, compared to 100% mortality of *T*. *castaneum* eggs, larvae, pupae, and adults without acclimation when exposed to 50°C for 35 min, respectively. The present results have important implications for developing successful heat treatment protocols to control *T*. *castaneum*, improving disinfestation effectiveness of heat treatment and understanding insect response to high temperatures.

## Introduction

The red flour beetle, *Tribolium castaneum* (Herbst) (Coleoptera: Tenebrionidae) ranks as one of the most destructive insects associated with food-processing facilities in the world. Fumigating food processing facilities with phosphine or methyl bromide has been a very effective method for controlling *T*. *castaneum* population for decades [1,2]. However, methyl bromide has been thoroughly phased out due to its ozone depleting potential according to the Montreal Protocol worldwide [3]. Meanwhile, intensive and repeated use of phosphine for control of *T*. *castaneum* population has resulted in extensive concerns regarding insecticide resistance, environmental contamination, pesticide residues, lethal effects on non-target organisms and so on [4–7]. Exploration and implementation of alternative control strategy and integrated pest management system have recently been considered to be the only solution to combat the increasing insecticide-resistant insect.

Heat treatment, as a potential alternative to methyl bromide for disinfesting mills and food-processing facilities, has been revisited to control stored-product insects [1, 8, 9]. Heat treatment, as an environment-friendly and convenient method, has been widely evaluated and implemented to effectively disinfest mills and food-processing facilities [10–13]. During the heat treatment, the target mill or food-processing facility usually is heated to 50–60°C which are then maintained for 24–36 h to completely eradicate stored product insects [14–17]. Naturally, the stored product insects inevitably experience acclimation to sublethal high temperature during gradually elevating temperature of the target mill or food-processing facility from ambient temperature to at least 50°C, which is the minimum effective temperature for disinfestation [17].

Although most of the researches have investigated the effect of high temperatures on mortality of stored grain insects [15,16,18–23], the influence of acclimation to sublethal temperatures on the mortality of *T*. *castaneum* is little known so far, which is very useful for developing successful heat treatment protocols for controlling *T*. *castaneum*, understanding insect response to high temperatures and the evolution of the reaction norms [24–27]. Here, our aim was to investigate if short-term acclimation to sublethal temperature affects heat tolerance of *T*. *castaneum* adults, pupae, larvae, and eggs when subsequently exposed to lethal high temperature of 50°C.

## Materials and methods

### Insects

Cultures of the *T*. *castaneum* were maintained in a controlled temperature and humidity chamber at 27±2°C, 75±5% r.h. and a 12:12 light:dark photoperiod without exposure to any pesticide at the Institute of Stored Product Insects of Henan University of Technology, Zhengzhou, China. The food media used were wheat flour and rolled oats (6:1, w/w). One-week-old adults, 1-d-old pupae, 18-d-old larvae, and 1-d-old eggs were randomly chosen for bioassays [16].

### Experimental protocol of acclimation to sublethal temperature

Twenty *T*. *castaneum* adults, pupae, larvae, and eggs were randomly selected and respectively put into empty plastic vials with a few of small holes for heat quick distribution, and then exposed to 36°C or 42°C [28, 29] in a temperature chamber (Thermocenter TN/GDW-010B, Tainuo Experiment Instrument Factory, Wuxi, Jiangsu, China) for 0 (control), 1, 5, 10, and 15 h as different acclimation periods, respectively. Subsequently, the acclimated *T*. *castaneum* adults, pupae, larvae, and eggs were respectively exposed to 50°C for 0, 10, 15, 20, 25, 30, and 35 min, and then the plastic vials holding adults or larvae were immediately opened. The treated adults or larvae in one plastic vial were gently brushed into a petri dish for determining their mortality. The adults or larvae were considered dead if no movement was observed when prodded with a camel’s hair brush. The plastic vials holding *T*. *castaneum* pupae were then maintained in insect culture environment, and the number of pupae developing into adults was recorded everyday for the following 7 days. The pupae that could not develop into adults were considered dead. The treated eggs in one plastic vial were gently brushed into a petri dish, and then maintained in insect culture environment. The number of eggs hatching into larvae was recorded everyday for the following 7 days. Three replicates were conducted. The acclimated *T*. *castaneum* adults, pupae, larvae, and eggs were respectively exposed to 50°C because it is the minimum effective temperature for facility heat treatment [17].

### RNA sequencing and quality control

Twenty *T*. *castaneum* larvae, pupae, and adults with acclimation to 42°C for 0 (control) and 10 h were flash-frozen in liquid N_2_ and stored at -80°C. Total RNA was isolated using Trizol Reagent (Invierogen, USA) according to the manufacturer’s instructions. An Agilent Bioanalyzer 2100 (Agilent Technologies) was used to assess the integrity/quality of the mRNA. Total RNA from the each treatment of *T*. *castaneum* larvae, pupae, and adults were respectively pooled for mRNA purification, and Illumina sequencing analysis was performed using a HiSeq 2500 with Beijing Biomarker Technologies CO., LTD (Beijing, China).

As a fundamental quality control measure, the Quality Score was used to evaluate base quality of the raw data. Clean reads were obtained by filtering the containing adapter or ploy-N and the low quality reads from raw data. The *T*. *castaneum* genome sequence was obtained from NCBI. Clean reads were aligned to the *T*. *castaneum* reference using TopHat 2.0 software. The relative expression levels of *hsp70*, *trpA1*, *painless* and *pyrexia* genes of *T*. *castaneum* larvae, pupae, and adults were determined using the fragments per kilobase of transcript per million mapped fragments method.

### Statistical analysis

The acclimated *T*. *castaneum* mortality after exposure to 50°C for different time intervals was calculated as a percentage. Mean ± SE mortality of the control *T*. *castaneum* adults, pupae, larvae, and eggs in all combinations of exposure temperature and exposure time was 0.00 ± 0.00, 1.11±1.11, 1.15±1.15, and 14.57±1.75%, respectively. Therefore, treatment mortality data in *T*. *castaneum* adults, pupae, and larvae were not corrected for control mortality, and treatment mortality data in *T*. *castaneum* eggs were corrected for control mortality [30]. Treatment percentage mortality was transformed to arcsine square-root value before subjecting to two-way analysis of variance (ANOVA) with insect mortality as response variable, and acclimation time, and exposure time as fixed effects. The mean mortality was compared and separated by Scheffe’s test at *p* = 0.05 level. These analyses were performed using SPSS Version 16.0 software [27].

## Results

### The mortality of *T*. *castaneum* eggs with acclimation to 36°C

**Fig 1:**
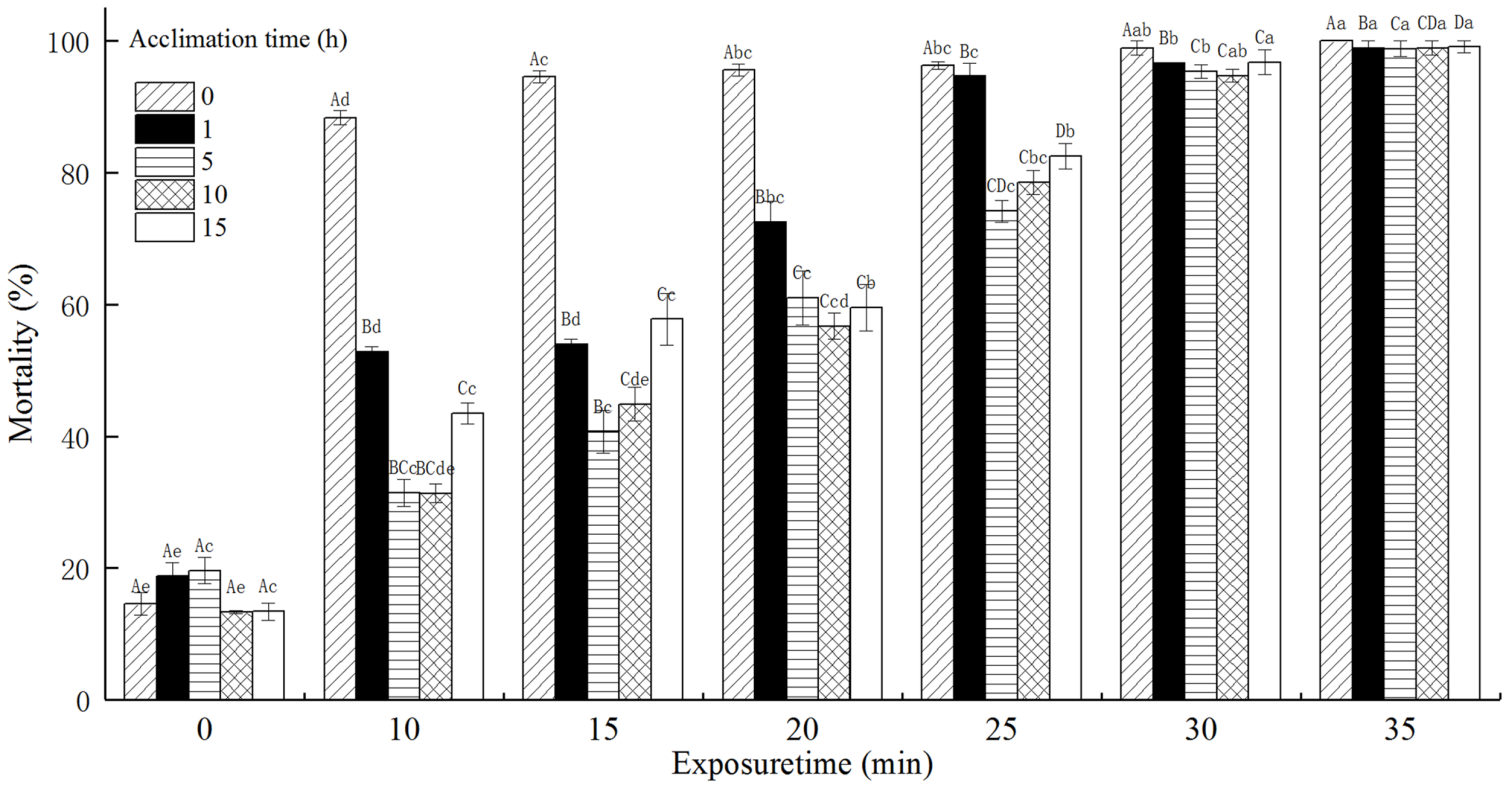
The effect of acclimation to 36°C on mortality (%) of *T*. *castaneum* eggs exposed to 50°C. Note: Different lowercase letters indicate significant differences at different exposure times for mortality (%) of *T*. *castaneum* with the same acclimation time, and different capital letters indicate significant differences at the same exposure time for mortality (%) of *T*. *castaneum* with different acclimation times (p<0.05). The same as below.

The mortality of *T*. *castaneum* eggs with acclimation to 36°C significantly increased with increasing exposure time when subsequently exposed to 50°C (Fig 1). Compared with the mortality of *T*. *castaneum* eggs without acclimation to 36°C (control), the mortality of acclimated *T*. *castaneum* eggs was significantly reduced when exposed to 50°C for the range of 0 to 25 min. The acclimation time, exposure time, and the interaction between the acclimation time and exposure time significantly affected the mortality of *T*. *castaneum* eggs at *p* < 0.05 level.

### The mortality of *T*. *castaneum* eggs with acclimation to 42°C

**Fig 2:**
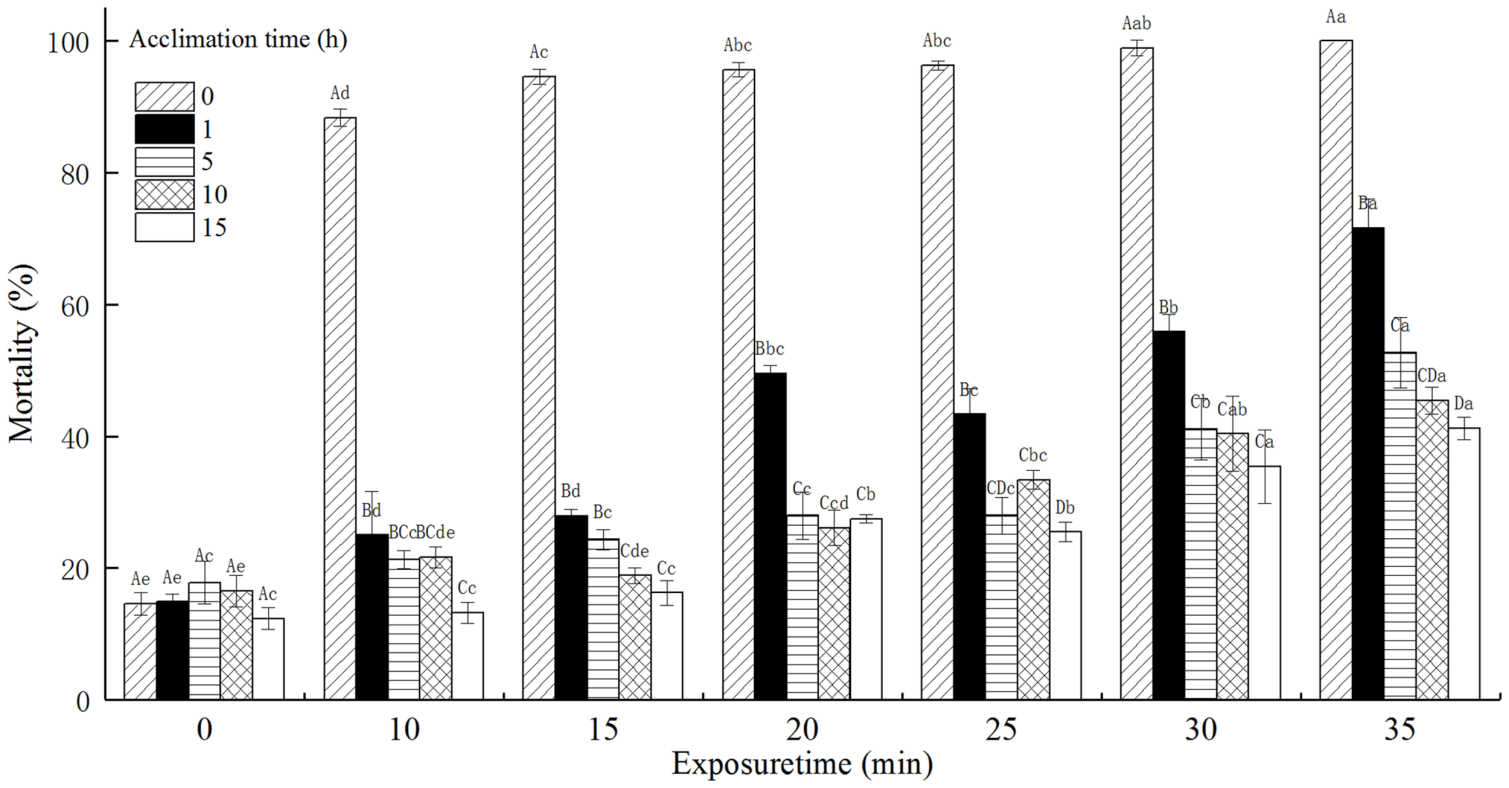
The effect of acclimation to 42°C on mortality (%) of *T*. *castaneum* eggs exposed to 50°C.

The mortality of *T*. *castaneum* eggs with acclimation to 42°C significantly increased with increasing exposure time when subsequently exposed to 50°C. The mortality of *T*. *castaneum* eggs significantly decreased with increasing acclimation time (Fig 2). Especially, the mortality of *T*. *castaneum* eggs without acclimation to 42°C (control) reached 100%, while the mortality of *T*. *castaneum* eggs with 15 h of acclimation to 42°C was only 41.24% when exposed to 50°C for 35 min. The acclimation time, exposure time, and the interaction between the acclimation time and exposure time significantly affected the mortality of *T*. *castaneum* eggs at *p* < 0.05 level.

### The mortality of *T*. *castaneum* larvae with acclimation to 36°C

**Fig 3:**
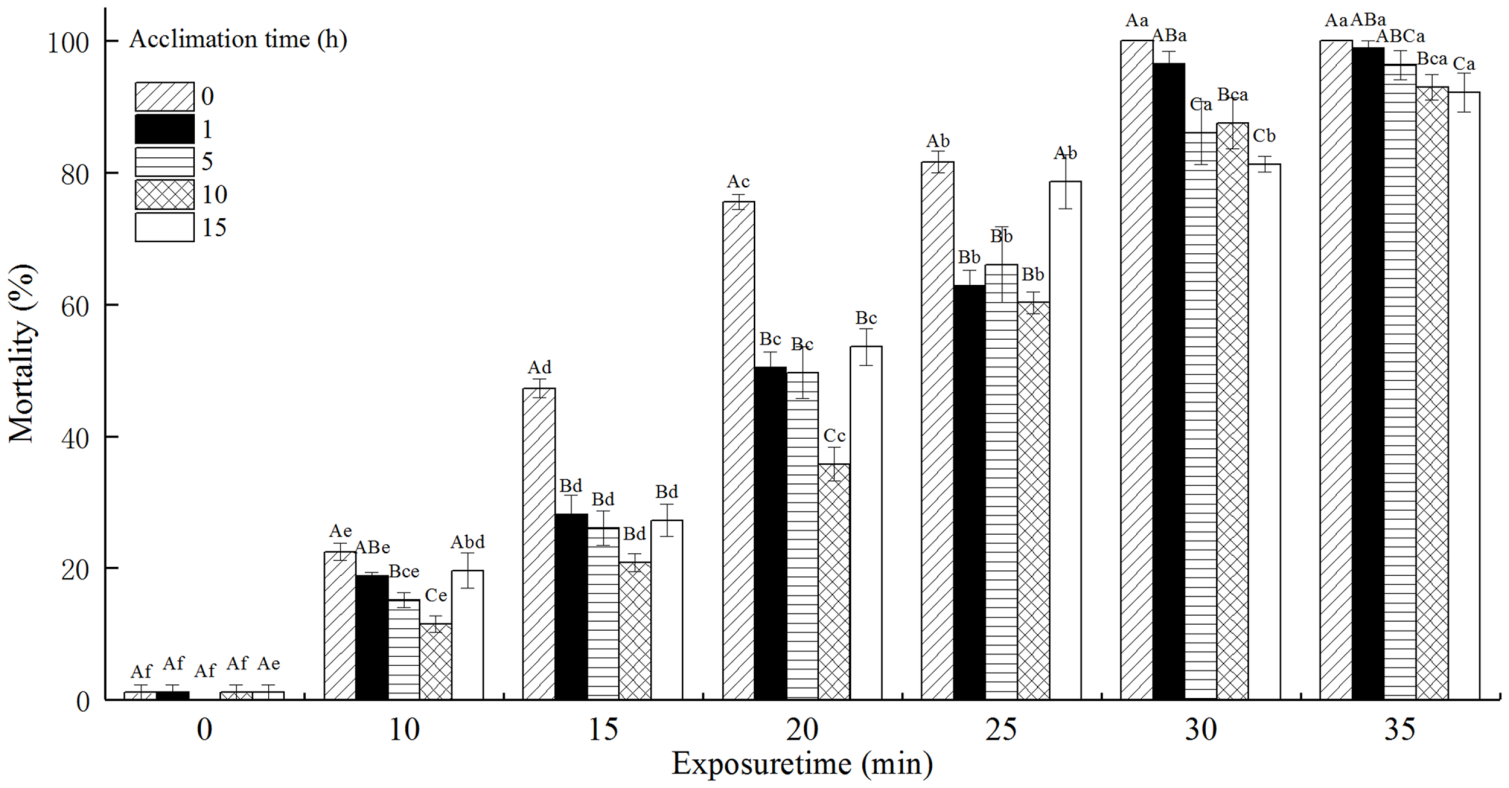
The effect of acclimation to 36°C on mortality (%) of *T*. *castaneum* larvae exposed to 50°C.

The mortality of *T*. *castaneum* larvae with acclimation to 36°C significantly increased with increasing exposure time when subsequently exposed to 50°C, and decreased with increasing acclimation time (Fig 3). Especially, the mortality of *T*. *castaneum* larvae without acclimation to 36°C (control) reached 100%, while the mortality of *T*. *castaneum* larvae with 15 h of acclimation to 36°C reached only 81.30% when exposed to 50°C for 30 min. The acclimation time, exposure time, and the interaction between the acclimation time and exposure time significantly affected the mortality of *T*. *castaneum* larvae at *p* < 0.05 level.

### The mortality of *T*. *castaneum* larvae with acclimation to 42°C

**Fig 4:**
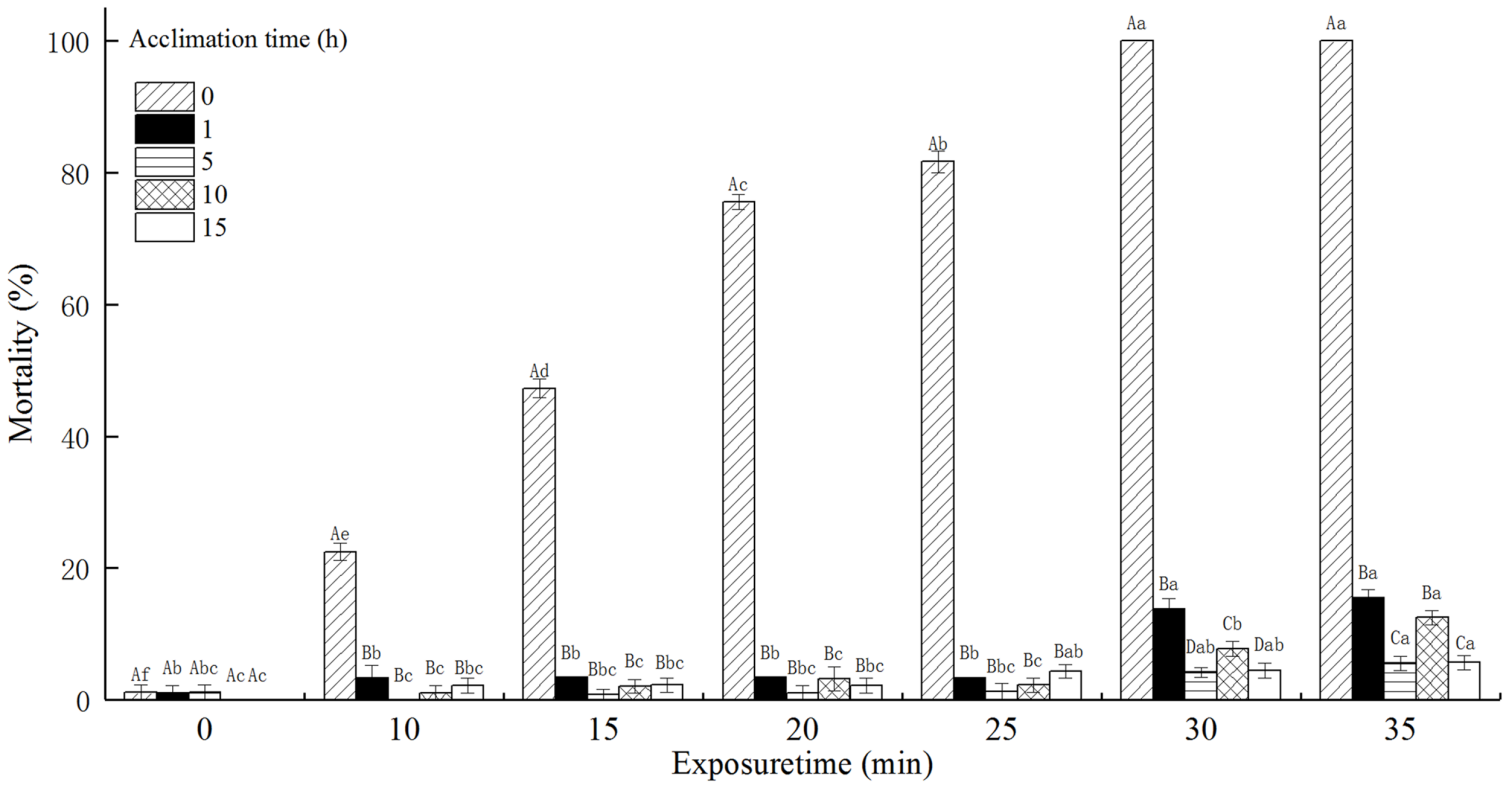
The effect of acclimation to 42°C on mortality (%) of *T*. *castaneum* larvae exposed to 50°C.

The mortality of *T*. *castaneum* larvae with acclimation to 42°C significantly increased with increasing exposure time when subsequently exposed to 50°C, and decreased with increasing acclimation time (Fig 4). Especially, the mortality of *T*. *castaneum* larvae without acclimation to 42°C (control) reached 100%, while the mortality of *T*. *castaneum* larvae with 1, 5, 10, and 15 h of acclimation to 42°C respectively reached only 13.85, 4.21, 7.78, and 4.44% when exposed to 50°C for 30 min. The acclimation time, exposure time, and the interaction between the acclimation time and exposure time significantly affected the mortality of *T*. *castaneum* larvae at *p* < 0.05 level.

### The mortality of *T*. *castaneum* pupae with acclimation to 36°C

**Fig 5:**
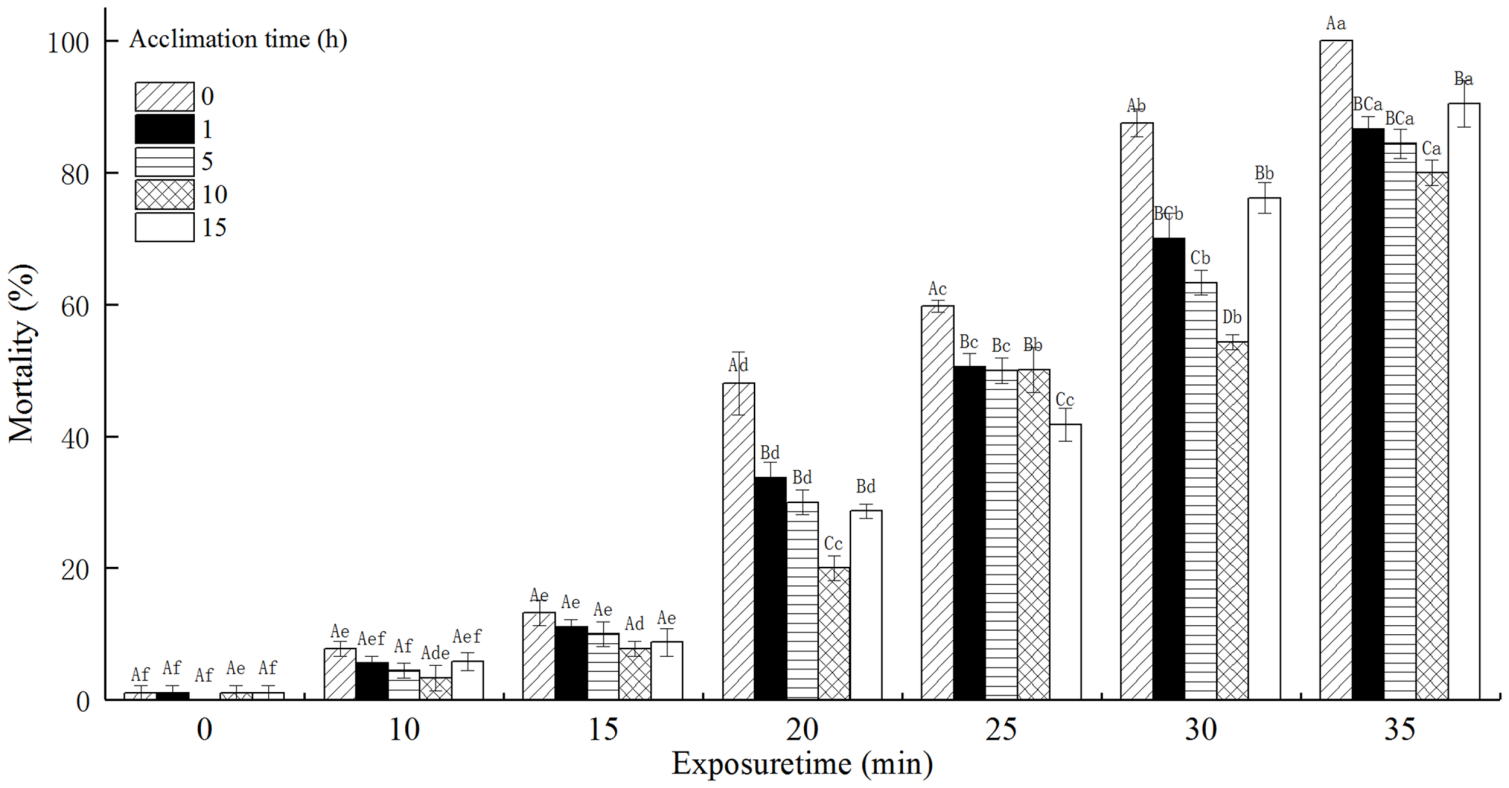
The effect of acclimation to 36°C on mortality (%) of *T*. *castaneum* pupae exposed to 50°C.

The mortality of *T*. *castaneum* pupae with acclimation to 36°C significantly increased with increasing exposure time when subsequently exposed to 50°C, and decreased with increasing acclimation time (Fig 5). Especially, the mortality of *T*. *castaneum* pupae without acclimation to 36°C (control) reached 100%, while the mortality of *T*. *castaneum* pupae with 10 h of acclimation to 36°C reached only 80.00% when exposed to 50°C for 35 min. The acclimation time, exposure time, and the interaction between the acclimation time and exposure time significantly affected the mortality of *T*. *castaneum* pupae at *p* < 0.05 level.

### The mortality of *T*. *castaneum* pupae with acclimation to 42°C

**Fig 6:**
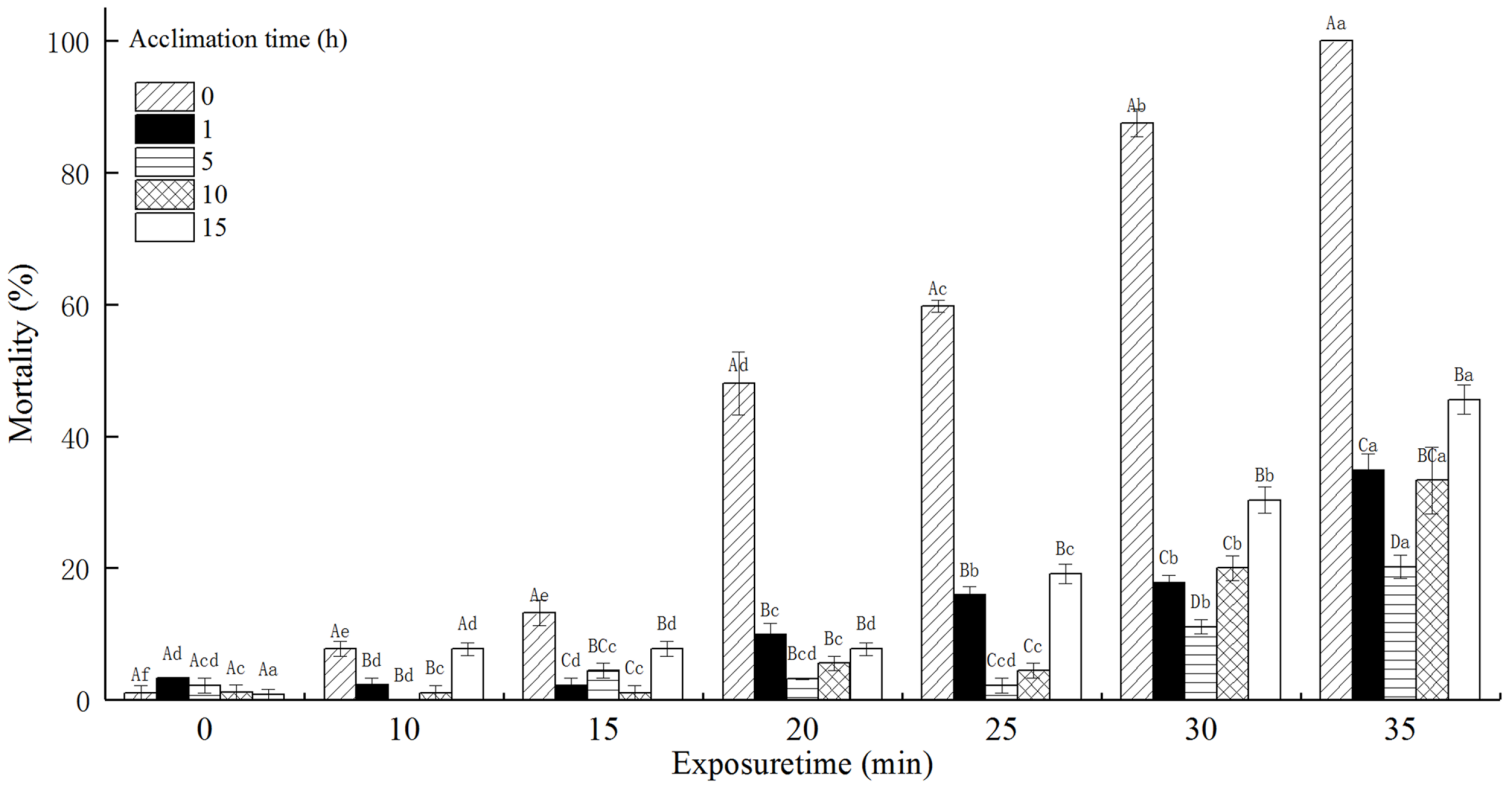
The effect of acclimation to 42°C on mortality (%) of *T*. *castaneum* pupae exposed to 50°C.

The mortality of *T*. *castaneum* pupae with acclimation to 42°C significantly increased with increasing exposure time when subsequently exposed to 50°C, and decreased with increasing acclimation time (Fig 6). Especially, the mortality of *T*. *castaneum* pupae without acclimation to 42°C (control) reached 100%, while the mortality of *T*. *castaneum* pupae with 1, 5, 10, and 15 h of acclimation to 42°C respectively reached only 34.87, 20.19, 33.33, and 45.56% when exposed to 50°C for 35 min. The acclimation time, exposure time, and the interaction between the acclimation time and exposure time significantly affected the mortality of *T*. *castaneum* pupae at *p* < 0.05 level.

### The mortality of *T*. *castaneum* adults with acclimation to 36°C

**Fig 7:**
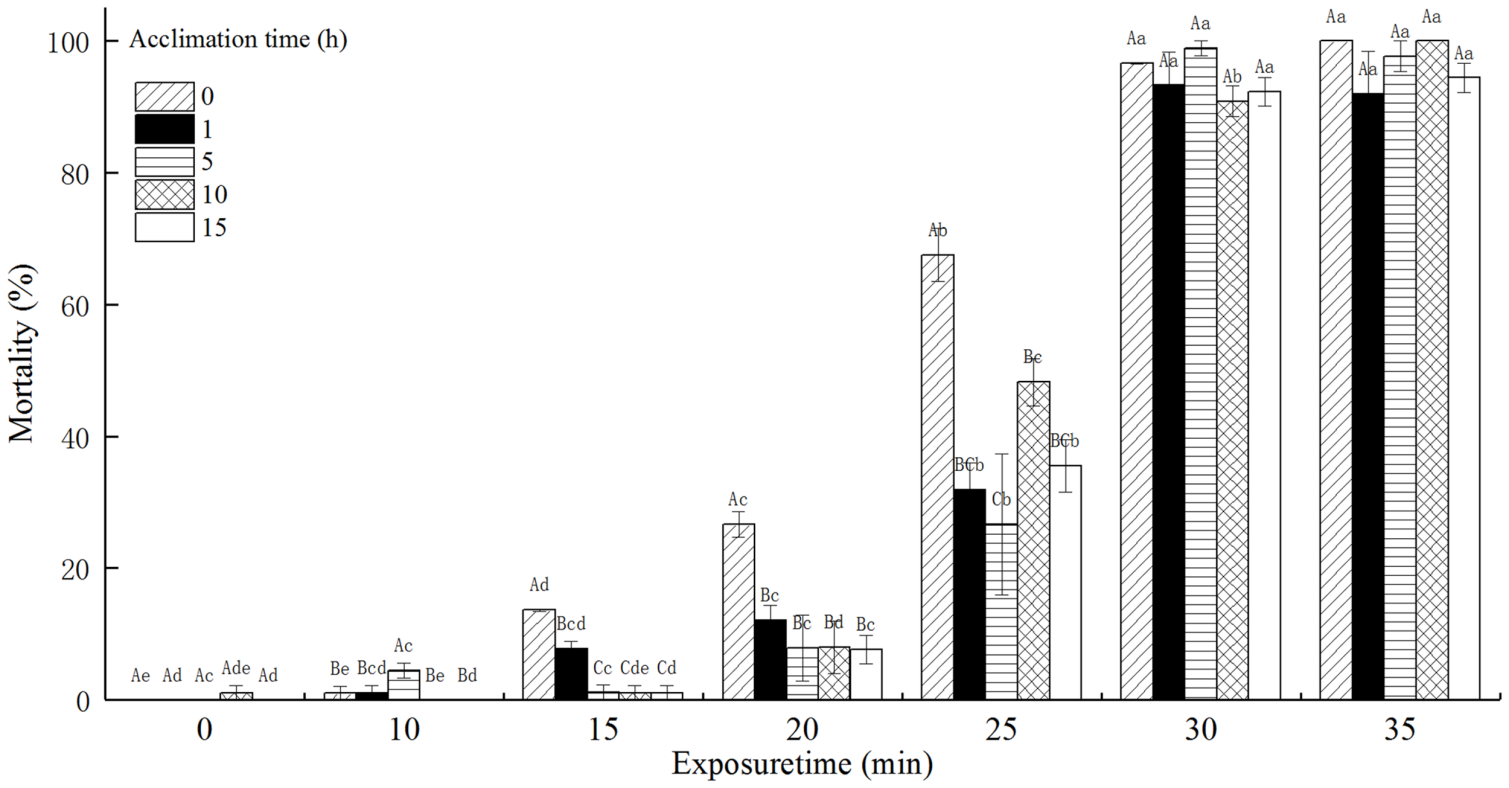
The effect of acclimation to 36°C on mortality (%) of *T*. *castaneum* adults exposed to 50°C.

The mortality of *T*. *castaneum* adults with acclimation to 36°C significantly increased with increasing exposure time, and decreased with increasing acclimation time, especially when subsequently exposed to 50°C from 0 to 25 min (Fig 7). Particularly, the mortality of *T*. *castaneum* adults without acclimation to 36°C (control) reached 67.56%, while the mortality of *T*. *castaneum* adults with 5 h of acclimation to 36°C reached only 26.67% when exposed to 50°C for 25 min. The acclimation time, exposure time, and the interaction between the acclimation time and exposure time significantly affected the mortality of *T*. *castaneum* adults at *p* < 0.05 level.

### The mortality of *T*. *castaneum* adults with acclimation to 42°C

**Fig 8:**
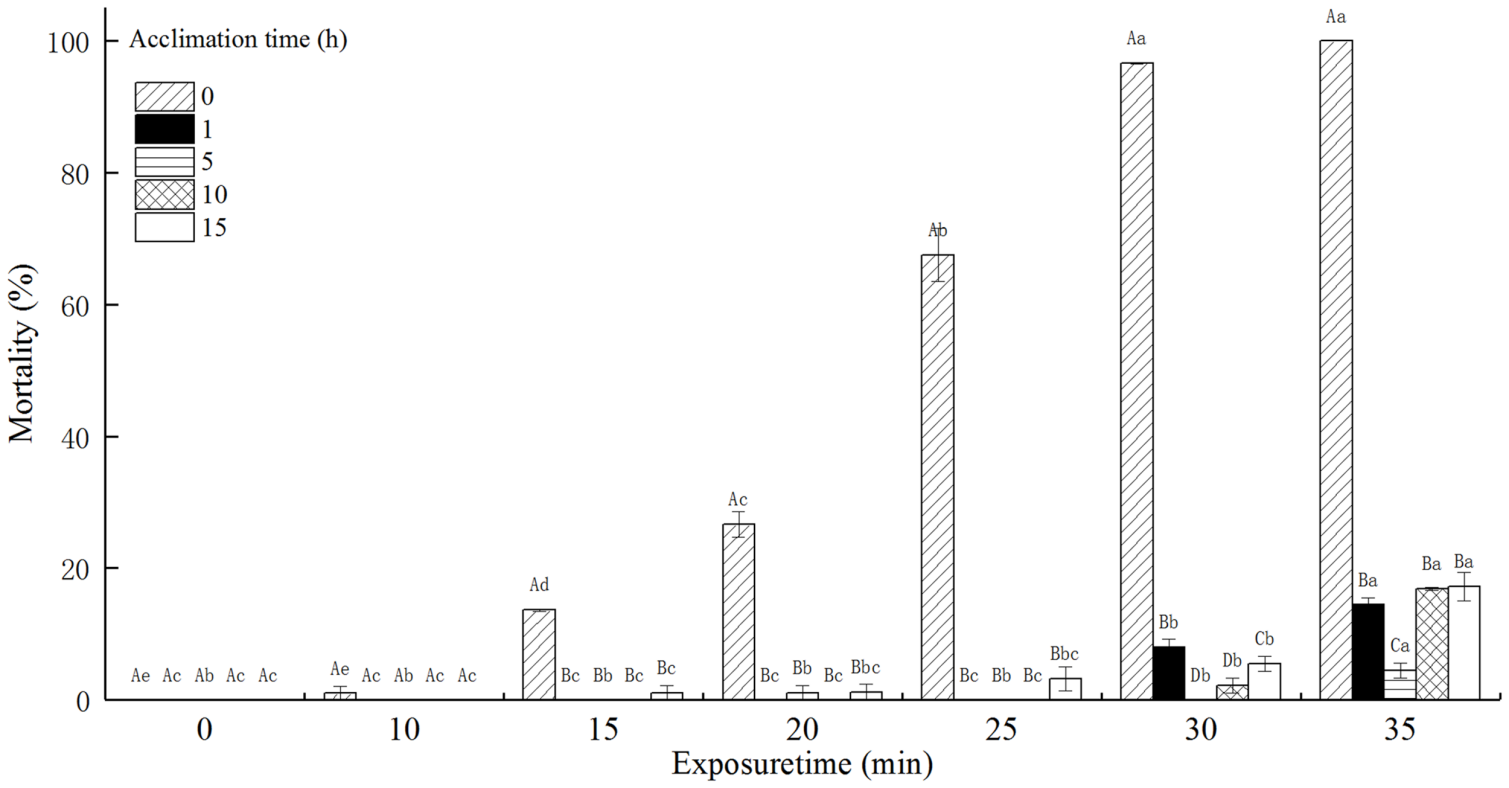
The effect of acclimation to 42°C on mortality (%) of *T*. *castaneum* adults exposed to 50°C.

The mortality of *T*. *castaneum* adults with acclimation to 42°C significantly increased with increasing exposure time when subsequently exposed to 50°C, and decreased with increasing acclimation time (Fig 8). Especially, the mortality of *T*. *castaneum* adults without acclimation to 42°C (control) reached 100%, while the mortality of *T*. *castaneum* adults with 1, 5, 10, and 15 h of acclimation to 42°C respectively reached only 14.44, 4.48, 16.86, and 17.20% when exposed to 50°C for 35 min. The acclimation time, exposure time, and the interaction between the acclimation time and exposure time significantly affected the mortality of *T*. *castaneum* adults at *p* < 0.05 level.

### The relative expression levels of *hsp70*, *trpA1*, *painless* and *pyrexia* genes of *T*. *castaneum*

**Fig 9:**
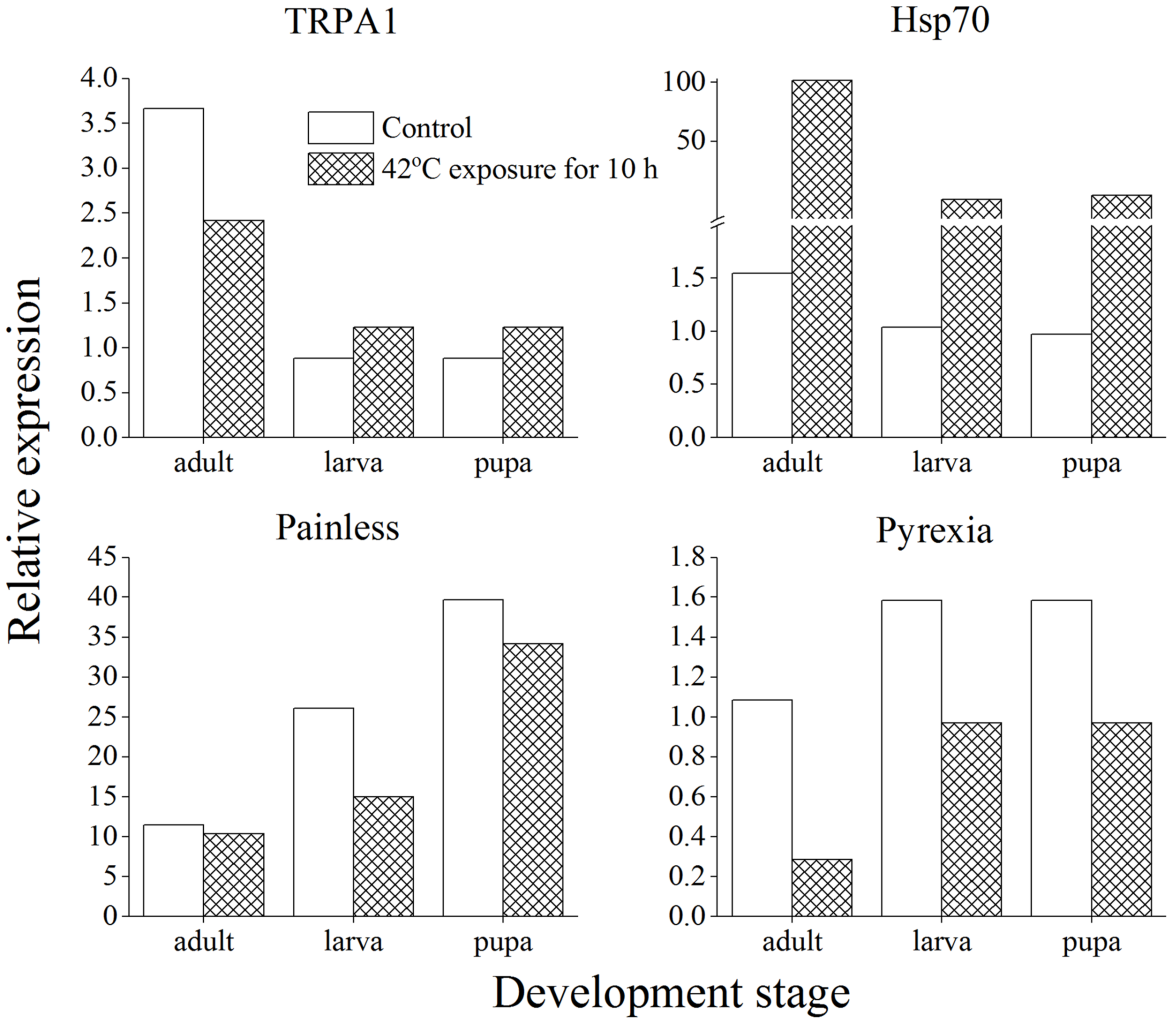
The relative expression of *hsp70*, *trpA1*, *painless* and *pyrexia* genes of *T*. *castaneum* with acclimation to 42°C for 10 h.

The Fig 9 shows that relative expression of *hsp70*, *trpA1*, *painless* and *pyrexia* genes of *T*. *castaneum* with acclimation to 42°C for 10 h. Compared to the controls, *hsp70* gene of *T*. *castaneum* larvae, pupae, and adults was significantly up-regulated after acclimation to 42°C for 10 h, the *trpA1* gene of *T*. *castaneum* larvae and pupae was also up-regulated, and the *painless* and *pyrexia* genes of *T*. *castaneum* was down-regulated.

## Discussion

The susceptibility of insects to lethal high temperatures was usually influenced by a variety of treatment factors, including insect strain, developmental stage, acclimation time, acclimation temperature, temperature-time combination, heating rate and so on [31, 32]. The present study results indicated that prior short-term acclimation to sublethal high temperatures of 36, and 42°C could significantly enhance the survival of *T*. *castaneum* eggs, larvae, pupae, and adults subsequently exposed to lethal high temperature of 50°C, and reduce their mortality. The mortality of *T*. *castaneum* eggs, larvae, pupae, and adults significantly increased with increasing exposure time at lethal high temperature of 50°C. Therefore, acclimation to sublethal high temperatures greatly enhanced the heat tolerance level of *T*. *castaneum* eggs, larvae, pupae, and adults, and significantly reduced their subsequent susceptibility to lethal high temperature.

Mahroof et al. (2003b) investigated time-mortality relationships for different life stages of *T*. *castaneum* exposed to constant temperatures of the range of 42 to 60°C [16]. Mortality of each life stage increased with increasing temperature and exposure time, and young larvae are the most heat-tolerant stage exposed to 50°C. The present study also gave the similar results. In addition, the present study results are also similar to Arthur (2006) [33] research results, which show that the mortality of late-instar larvae, pupae, and adults of *T*. *castaneum* and *Tribolium confusum* in gradually increasing temperature conditions was lower than that in suddenly increasing temperature conditions. The difference is that the *T*. *castaneum* was acclimated at constant temperatures in the current study, whereas the *T*. *castaneum* is exposed in gradually increasing temperature conditions in Arthur (2006) [33] research. The present results as well as the previous research results are very helpful to understand insect response to different high temperature treatments, in favor of designing effective heat treatment protocols for controlling *T*. *castaneum* in practice.

In the current study, the influence of short term acclimation of *T*. *castaneum* eggs, larvae, pupae, and adults to sublethal temperature of 42°C on their subsequent susceptibility to lethal high temperature of 50°C was apparently greater than that of acclimation to 36°C. The most influential acclimation times to 42°C for mortality of *T*. *castaneum* eggs, larvae, pupae, and adults were 15, 5, 5, and 5 h, respectively, and the most influential acclimation times to 36°C for mortality of *T*. *castaneum* eggs, larvae, pupae, and adults were 5, 10, 10, and 15 h, respectively. The influence of acclimation time and developmental stage on the heat tolerance level of *T*. *castaneum* was variable. This may be related to the ability of different developmental stage insects to deal with different high temperature stress, which deserves to be further investigated.

The ability of insects to cope with high temperature stress can be achieved by physiological and biochemical mechanisms [34–36]. Emekci et al. (2002) [37] showed that respiration of *T*. *castaneum* young larvae is significantly higher than other developmental stages, which may result in higher metabolic rates often associated with a response to stress and may enhance adaption to unfavorable conditions. Insects also can alter their sensitivity to heat stress through short-term acclimation or long-term evolutionary adaptation [38]. Short-term heat acclimation in laboratory may involve physiological and biochemical changes in insects, and then change their responses to the ambient variable environmental temperatures [39–41]. Three different *hsp70* genes, *tchsp70 I*, *tchsp70 II*, and *tchsp70 III*, respectively encode a heat-inducible HSP, a constitutively expressed HSP, and a developmentally regulated HSP in *T*. *castaneum* young larvae which are responsible for enhancing heat tolerance level [42]. The *trpA1* is closely associated with high temperature sensing and also increasing high-temperature tolerance, the *painless* is responsible for rapid acclimation to high temperature, and the *pyrexia* plays an important role in protecting *T*. *castaneum* adults from acute heat stress. RNAi bioassay results also showed that relatively short exposure to high temperature (1 min at 52°C or 10 min at 42°C) was enough to give rise to thermal acclimation [29]. Heat shock increases three heat shock protein (hsp) genes *hsps—Szhsp70*, *Szhsc70*, and *Szhsp90* expression in *S*. *zeamais* with the highest upregulation at 40°C, and the intensity of upregulation is ranked as follows: *Szhsp70* > *Szhsp90* > *Szhsc70* [43]. The present results indicated that *hsp70* and *trpA1* genes of *T*. *castaneum* were up-regulated after acclimation to 42°C for 10 h. Therefore, the *hsps* as well as some other physiological and biochemical adaptation mechanisms, were involved in the heat tolerance of *T*. *castaneum* with acclimation to sublethel high temperatures for varying periods. Detailed genetic expression variation and metabolic pathways associated with acclimation of *T*. *castaneum* eggs, larvae, pupae, and adults to sublethel high temperatures deserve to be further investigated, which are beneficial to understand insects responses to thermal stress and their adaptation evolution mechanisms in response to ongoing climate warming [44].

Usually, the temperatures don’t increase at the same rate, and not evenly distribute at different portions of the whole facility during heat treatment, which inevitably makes the stored product insects experience the acclimation to sublethal temperatures. The present research simulated discrete heating condition in some portions by exposing the insects to sublethal temperatures for a short period. The results clearly indicated that short-term acclimation to sublethal temperatures enhanced their ability of adaption to the high temperature stress environment, and significantly reduced subsequent heat susceptibility of *T*. *castaneum* exposed to 50°C. Therefore, the temperatures of the different portions in the whole target facility should be increased to more than 50°C at the quickest rate, which will avoid the acclimation of stored product insects to sublethal temperatures to the maximum extent during implementing heat treatment, and enhance efficacy of heat treatment in practice.

## Supporting information

S1 Table The effect of acclimation to 36°C on mortality (%) of *T*. *castaneum* eggs exposed to 50°C. (DOCX)

S2 Table Two way analysis of variance (ANOVA) parameters for main effects and associated interactions for the mortality of *T*. *castaneum* eggs with acclimation to 36°C exposed to 50°C. (DOCX)

S3 Table The effect of acclimation to 42°C on mortality (%) of *T*. *castaneum* eggs exposed to 50°C. (DOCX)

S4 Table Two way analysis of variance (ANOVA) parameters for main effects and associated interactions for the mortality of *T*. *castaneum* eggs with acclimation to 42°C exposed to 50°C. (DOCX)

S5 Table The effect of acclimation to 36°C on mortality (%) of *T*. *castaneum* larvae exposed to 50°C. (DOCX)

S6 Table Two way analysis of variance (ANOVA) parameters for main effects and associated interactions for the mortality of *T*. *castaneum* larvae with acclimation to 36°C exposed to 50°C. (DOCX)

S7 Table The effect of acclimation to 42°C on mortality (%) of *T*. *castaneum* larvae exposed to 50°C. (DOCX)

S8 Table Two way analysis of variance (ANOVA) parameters for main effects and associated interactions for the mortality of *T*. *castaneum* larvae with acclimation to 42°C exposed to 50°C. (DOCX)

S9 Table The effect of acclimation to 36°C on mortality (%) of *T*. *castaneum* pupae exposed to 50°C. (DOCX)

S10 Table Two way analysis of variance (ANOVA) parameters for main effects and associated interactions for the mortality of *T*. *castaneum* pupae with acclimation to 36°C exposed to 50°C. (DOCX)

S11 Table The effect of acclimation to 42°C on mortality (%) of *T*. *castaneum* pupae exposed to 50°C. (DOCX)

S12 Table Two way analysis of variance (ANOVA) parameters for main effects and associated interactions for the mortality of *T*. *castaneum* pupae with acclimation to 42°C exposed to 50°C. (DOCX)

S13 Table The effect of acclimation to 36°C on mortality (%) of *T*. *castaneum* adults exposed to 50°C. (DOCX)

S14 Table Two way analysis of variance (ANOVA) parameters for main effects and associated interactions for the mortality of *T*. *castaneum* adults with acclimation to 36°C exposed to 50°C. (DOCX)

S15 Table The effect of acclimation to 42°C on mortality (%) of *T*. *castaneum* adults exposed to 50°C. (DOCX)

S16 Table Two way analysis of variance (ANOVA) parameters for main effects and associated interactions for the mortality of *T*. *castaneum* adults with acclimation to 42°C exposed to 50°C. (DOCX)
