# Supplementary material for: Influence of acclimation to sublethal temperature on heat tolerance of Tribolium castaneum (Herbst) (Coleoptera: Tenebrionidae) exposed to 50°C
Source: PLoS One. 2017 Aug 7;12(8):e0182269. doi: 10.1371/journal.pone.0182269 (PMC5546633; doi:10.1371/journal.pone.0182269)
Supplement: S1 Table — (DOCX) [file pone.0182269.s001.docx]

S1 Table The effect of acclimation to 36℃ on mortality (%) of *T. castaneum* eggs exposed to 50℃

| Exposure time /min | Acclimation time /h | | | | |
| --- | --- | --- | --- | --- | --- |
|  | 0 | 1 | 5 | 10 | 15 |
| 0 | 14.57±1.75BCe | 18.81±2.05ABd | 19.65±2.03Af | 13.34±0.26Cf | 13.42±1.30BCe |
| 10 | 88.38±1.09Ad | 52.87±0.71Bc | 31.44±2.03De | 31.35±1.43De | 43.51±1.58Cd |
| 15 | 94.58±0.93Ac | 54.00±0.78Bc | 40.72±3.25Cd | 44.90±2.61Cd | 57.79±3.90Bc |
| 20 | 95.62±0.93Abc | 72.52±3.22Bb | 61.04±4.07Cc | 56.76±1.99Cc | 59.55±3.58Cc |
| 25 | 96.29±0.59Abc | 94.65±2.01Aa | 74.21±1.66Cb | 78.55±1.78BCb | 82.51±1.96Bb |
| 30 | 98.96±1.04Aab | 96.66±0.06Aa | 95.39±1.03Aa | 94.73±0.96Aa | 96.77±1.86Aa |
| 35 | 100.00±0.00Aa | 98.96±1.04Aa | 98.85±1.15Aa | 98.96±1.04Aa | 99.10±0.90Aa |

Note: Data are Mean ± SE of three replicates. Different lowercase letters indicate significant differences in the same column, and different capital letters indicate significant differences in the same row（p<0.05）. The same as below.
