# Supplementary material for: Influence of acclimation to sublethal temperature on heat tolerance of Tribolium castaneum (Herbst) (Coleoptera: Tenebrionidae) exposed to 50°C
Source: PLoS One. 2017 Aug 7;12(8):e0182269. doi: 10.1371/journal.pone.0182269 (PMC5546633; doi:10.1371/journal.pone.0182269)
Supplement: S3 Table — (DOCX) [file pone.0182269.s003.docx]

S3 Table The effect of acclimation to 42℃ on mortality (%) of *T. castaneum* eggs exposed to 50℃

| Exposure time /min | Acclimation time /h | | | | |
| --- | --- | --- | --- | --- | --- |
|  | 0 | 1 | 5 | 10 | 15 |
| 0 | 14.57±1.75Ae | 14.90±0.97Ae | 17.81±2.76Ac | 16.52±2.05Ae | 12.33±1.42Ac |
| 10 | 88.38±1.09Ad | 25.00±5.70Bd | 21.33±1.19BCc | 21.65±1.32BCde | 13.28±1.37Cc |
| 15 | 94.58±0.93Ac | 27.95±0.81Bd | 24.34±1.29Bc | 18.89±1.00Cde | 16.25±1.58Cc |
| 20 | 95.62±0.93Abc | 49.56±1.08Bbc | 27.98±3.05Cc | 26.11±2.27Ccd | 27.50±0.56Cb |
| 25 | 96.29±0.59Abc | 43.34±3.36Bc | 28.00±2.37CDc | 33.42±1.17Cbc | 25.47±1.26Db |
| 30 | 98.96±1.04Aab | 55.84±2.28Bb | 41.11±4.01Cb | 40.38±4.85Cab | 35.42±4.74Ca |
| 35 | 100.00±0.00Aa | 71.58±3.75Ba | 52.68±4.58Ca | 45.45±1.75CDa | 41.24±1.45Da |
