# Supplementary material for: Influence of acclimation to sublethal temperature on heat tolerance of Tribolium castaneum (Herbst) (Coleoptera: Tenebrionidae) exposed to 50°C
Source: PLoS One. 2017 Aug 7;12(8):e0182269. doi: 10.1371/journal.pone.0182269 (PMC5546633; doi:10.1371/journal.pone.0182269)
Supplement: S5 Table — (DOCX) [file pone.0182269.s005.docx]

S5 Table The effect of acclimation to 36℃ on mortality (%) of *T. castaneum* larvae exposed to 50℃

| Exposure time /min | Acclimation time /h | | | | |
| --- | --- | --- | --- | --- | --- |
|  | 0 | 1 | 5 | 10 | 15 |
| 0 | 1.15±1.15Af | 1.15±1.15Af | 0.00±0.00Af | 1.15±1.15Af | 1.15±1.15Ae |
| 10 | 22.49±1.27Ae | 18.79±0.63ABe | 15.13±1.14BCe | 11.53±1.21Ce | 19.64±2.69ABd |
| 15 | 47.28±1.43Ad | 28.16±2.89Bd | 26.09±2.60Bd | 20.85±1.39Bd | 27.24±2.43Bd |
| 20 | 75.56±1.11Ac | 50.42±2.44Bc | 49.70±3.89Bc | 35.79±2.57Cc | 53.60±2.79Bc |
| 25 | 81.68±1.65Ab | 62.87±2.33Bb | 66.05±5.78Bb | 60.27±1.69Bb | 78.62±4.09Ab |
| 30 | 100.00±0.00Aa | 96.47±1.99ABa | 86.05±4.81Ca | 87.53±3.84BCa | 81.30±1.16Cb |
| 35 | 100.00±0.00Aa | 98.92±1.08ABa | 96.36±2.23ABCa | 93.02±1.92BCa | 92.18±2.95Ca |
