# Supplementary material for: Influence of acclimation to sublethal temperature on heat tolerance of Tribolium castaneum (Herbst) (Coleoptera: Tenebrionidae) exposed to 50°C
Source: PLoS One. 2017 Aug 7;12(8):e0182269. doi: 10.1371/journal.pone.0182269 (PMC5546633; doi:10.1371/journal.pone.0182269)
Supplement: S7 Table — (DOCX) [file pone.0182269.s007.docx]

S7 Table The effect of acclimation to 42℃ on mortality (%) of *T. castaneum* larvae exposed to 50℃

| Exposure time /min | Acclimation time /h | | | | |
| --- | --- | --- | --- | --- | --- |
|  | 0 | 1 | 5 | 10 | 15 |
| 0 | 1.15±1.15Af | 1.11±1.11Ab | 1.15±1.15Abc | 0.00±0.00Ac | 0.00±0.00Ac |
| 10 | 22.49±1.27Ae | 3.33±1.92Bb | 0.00±0.00Bc | 1.08±1.08Bc | 2.22±1.11Bbc |
| 15 | 47.28±1.43Ad | 3.41±0.04Bb | 0.83±0.83Bbc | 2.06±1.04Bc | 2.26±1.13Bbc |
| 20 | 75.56±1.11Ac | 3.41±0.08Bb | 1.11±1.11Bbc | 3.19±1.81Bc | 2.22±1.11Bbc |
| 25 | 81.68±1.65Ab | 3.37±0.04Bb | 1.28±1.28Bbc | 2.26±1.13Bc | 4.37±1.04Bab |
| 30 | 100.00±0.00Aa | 13.85±1.50Ba | 4.21±0.76Dab | 7.78±1.11Cb | 4.44±1.11Dab |
| 35 | 100.00±0.00Aa | 15.57±1.16Ba | 5.59±1.07Ca | 12.49±1.08Ba | 5.67±1.11Ca |
