# Supplementary material for: Influence of acclimation to sublethal temperature on heat tolerance of Tribolium castaneum (Herbst) (Coleoptera: Tenebrionidae) exposed to 50°C
Source: PLoS One. 2017 Aug 7;12(8):e0182269. doi: 10.1371/journal.pone.0182269 (PMC5546633; doi:10.1371/journal.pone.0182269)
Supplement: S9 Table — (DOCX) [file pone.0182269.s009.docx]

S9 Table The effect of acclimation to 36℃ on mortality (%) of *T. castaneum* pupae exposed to 50℃

| Exposure time /min | Acclimation time /h | | | | |
| --- | --- | --- | --- | --- | --- |
|  | 0 | 1 | 5 | 10 | 15 |
| 0 | 1.11±1.11Af | 1.11±1.11Af | 0.00±0.00Af | 1.11±1.11Ae | 1.08±1.08Af |
| 10 | 7.78±1.11Ae | 5.56±1.11Aef | 4.44±1.11Af | 3.37±1.92Ade | 5.83±1.33Aef |
| 15 | 13.19±1.93Ae | 11.11±1.11Ae | 10.00±1.92Ae | 7.78±1.11Ad | 8.75±2.08Ae |
| 20 | 48.08±4.75Ad | 33.75±2.30Bd | 30.00±1.92Bd | 20.00±1.92Cc | 28.67±1.07Bd |
| 25 | 59.78±0.94Ac | 50.57±2.01Bc | 50.00±1.92Bc | 50.08±3.37Bb | 41.79±2.47Cc |
| 30 | 87.54±2.11Ab | 70.00±3.85BCb | 63.33±1.92Cb | 54.31±1.18Db | 76.18±2.36Bb |
| 35 | 100.00±0.00Aa | 86.67±1.92BCa | 84.44±2.22BCa | 80.00±1.92Ca | 90.45±3.55Ba |
