# Supplementary material for: Influence of acclimation to sublethal temperature on heat tolerance of Tribolium castaneum (Herbst) (Coleoptera: Tenebrionidae) exposed to 50°C
Source: PLoS One. 2017 Aug 7;12(8):e0182269. doi: 10.1371/journal.pone.0182269 (PMC5546633; doi:10.1371/journal.pone.0182269)
Supplement: S11 Table — (DOCX) [file pone.0182269.s011.docx]

S11 Table The effect of acclimation to 42℃ on mortality (%) of *T. castaneum* pupae exposed to 50℃

| Exposure time /min | Acclimation time /h | | | | |
| --- | --- | --- | --- | --- | --- |
|  | 0 | 1 | 5 | 10 | 15 |
| 0 | 1.11±1.11Af | 3.30±0.04Ad | 2.22±1.11Acd | 1.15±1.15Ac | 0.83±0.83Aa |
| 10 | 7.78±1.11Ae | 2.26±1.13Bd | 0.00±0.00Bd | 1.11±1.11Bc | 7.75±0.97Ad |
| 15 | 13.19±1.93Ae | 2.22±1.11Cd | 4.44±1.11BCc | 1.11±1.11Cc | 7.78±1.11Bd |
| 20 | 48.08±4.75Ad | 9.86±1.80Bc | 3.23±0.11Bcd | 5.56±1.11Bc | 7.75±0.97Bd |
| 25 | 59.78±0.94Ac | 15.91±1.29Bb | 2.22±1.11Ccd | 4.44±1.11Cc | 19.14±1.50Bc |
| 30 | 87.54±2.11Ab | 17.78±1.11Cb | 11.11±1.11Db | 20.00±1.92Cb | 30.34±1.96Bb |
| 35 | 100.00±0.00Aa | 34.87±2.46Ca | 20.19±1.76Da | 33.33±5.09BCa | 45.56±2.22Ba |
