# Supplementary material for: Influence of acclimation to sublethal temperature on heat tolerance of Tribolium castaneum (Herbst) (Coleoptera: Tenebrionidae) exposed to 50°C
Source: PLoS One. 2017 Aug 7;12(8):e0182269. doi: 10.1371/journal.pone.0182269 (PMC5546633; doi:10.1371/journal.pone.0182269)
Supplement: S12 Table — (DOCX) [file pone.0182269.s012.docx]

S12 Table Two way analysis of variance (ANOVA) parameters for main effects and associated interactions for the mortality of *T. castaneum* pupae with acclimation to 42℃ exposed to 50℃

| Source | df | Type III SS | Mean square | F-value | p-value |
| --- | --- | --- | --- | --- | --- |
| Acclimation time | 4 | 2.093 | 0.523 | 567.508 | < 0.001 |
| Exposure time | 6 | 2.544 | 0.424 | 459.921 | < 0.001 |
| Acclimation time × Exposure time | 24 | 1.336 | 0.056 | 60.381 | < 0.001 |
| Error | 70 | 0.065 | 0.001 |  |  |
| Total | 105 | 9.457 |  |  |  |
