# Supplementary material for: Influence of acclimation to sublethal temperature on heat tolerance of Tribolium castaneum (Herbst) (Coleoptera: Tenebrionidae) exposed to 50°C
Source: PLoS One. 2017 Aug 7;12(8):e0182269. doi: 10.1371/journal.pone.0182269 (PMC5546633; doi:10.1371/journal.pone.0182269)
Supplement: S13 Table — (DOCX) [file pone.0182269.s013.docx]

S13 Table The effect of acclimation to 36℃ on mortality (%) of *T. castaneum* adults exposed to 50℃

| Exposure time /min | Acclimation time /h | | | | |
| --- | --- | --- | --- | --- | --- |
|  | 0 | 1 | 5 | 10 | 15 |
| 0 | 0.00±0.00Ae | 0.00±0.00Ad | 0.00±0.00Ac | 1.11±1.11Ade | 0.00±0.00Ad |
| 10 | 1.01±1.01Be | 1.11±1.11Bcd | 4.44±1.11Ac | 0.00±0.00Be | 0.00±0.00Bd |
| 15 | 13.67±0.17Ad | 7.78±1.11Bcd | 1.15±1.15Cc | 1.11±1.11Cde | 1.11±1.11Cd |
| 20 | 26.67±1.92Ac | 12.11±2.28Bc | 7.90±5.03Bc | 8.00±4.04Bd | 7.67±2.17Bc |
| 25 | 67.56±4.09Ab | 31.90±4.11BCb | 26.67±10.72Cb | 48.24±3.56Bc | 35.56±4.01BCb |
| 30 | 96.66±0.13Aa | 93.30±5.08Aa | 98.89±1.11Aa | 90.88±2.34Ab | 92.33±2.17Aa |
| 35 | 100.00±0.00Aa | 91.99±6.41Aa | 97.70±2.30Aa | 100.00±0.00Aa | 94.44±2.22Aa |
