# Supplementary material for: Influence of acclimation to sublethal temperature on heat tolerance of Tribolium castaneum (Herbst) (Coleoptera: Tenebrionidae) exposed to 50°C
Source: PLoS One. 2017 Aug 7;12(8):e0182269. doi: 10.1371/journal.pone.0182269 (PMC5546633; doi:10.1371/journal.pone.0182269)
Supplement: S14 Table — (DOCX) [file pone.0182269.s014.docx]

S14 Table Two way analysis of variance (ANOVA) parameters for main effects and associated interactions for the mortality of *T. castaneum* adults with acclimation to 36℃ exposed to 50℃

| Source | df | Type III SS | Mean square | F-value | p-value |
| --- | --- | --- | --- | --- | --- |
| Acclimation time | 4 | 0.159 | 0.040 | 13.757 | < 0.001 |
| Exposure time | 6 | 16.735 | 2.789 | 962.735 | < 0.001 |
| Acclimation time × Exposure time | 24 | 0.310 | 0.013 | 4.460 | < 0.001 |
| Error | 70 | 0.203 | 0.003 |  |  |
| Total | 105 | 31.015 |  |  |  |
