# Supplementary material for: Influence of acclimation to sublethal temperature on heat tolerance of Tribolium castaneum (Herbst) (Coleoptera: Tenebrionidae) exposed to 50°C
Source: PLoS One. 2017 Aug 7;12(8):e0182269. doi: 10.1371/journal.pone.0182269 (PMC5546633; doi:10.1371/journal.pone.0182269)
Supplement: S15 Table — (DOCX) [file pone.0182269.s015.docx]

S15 Table The effect of acclimation to 42℃ on mortality (%) of *T. castaneum* adults exposed to 50℃

| Exposure time /min | Acclimation time /h | | | | |
| --- | --- | --- | --- | --- | --- |
|  | 0 | 1 | 5 | 10 | 15 |
| 0 | 0.00±0.00Ae | 0.00±0.00Ac | 0.00±0.00Ab | 0.00±0.00Ac | 0.00±0.00Ac |
| 10 | 1.01±1.01Ae | 0.00±0.00Ac | 0.00±0.00Ab | 0.00±0.00Ac | 0.00±0.00Ac |
| 15 | 13.67±0.17Ad | 0.00±0.00Bc | 0.00±0.00Bb | 0.00±0.00Bc | 1.08±1.08Bc |
| 20 | 26.67±1.92Ac | 0.00±0.00Bc | 1.08±1.08Bb | 0.00±0.00Bc | 1.19±1.19Bbc |
| 25 | 67.56±4.09Ab | 0.00±0.00Bc | 0.00±0.00Bb | 0.00±0.00Bc | 3.23±1.81Bbc |
| 30 | 96.66±0.13Aa | 8.05±1.15Bb | 0.00±0.00Db | 2.19±1.09Db | 5.49±1.19Cb |
| 35 | 100.00±0.00Aa | 14.44±1.11Ba | 4.48±1.09Ca | 16.86±0.19Ba | 17.20±2.15Ba |
