# Supplementary material for: Influence of acclimation to sublethal temperature on heat tolerance of Tribolium castaneum (Herbst) (Coleoptera: Tenebrionidae) exposed to 50°C
Source: PLoS One. 2017 Aug 7;12(8):e0182269. doi: 10.1371/journal.pone.0182269 (PMC5546633; doi:10.1371/journal.pone.0182269)
Supplement: S16 Table — (DOCX) [file pone.0182269.s016.docx]

S16 Table Two way analysis of variance (ANOVA) parameters for main effects and associated interactions for the mortality of *T. castaneum* adults with acclimation to 42℃ exposed to 50℃

| Source | df | Type III SS | Mean square | F-value | p-value |
| --- | --- | --- | --- | --- | --- |
| Acclimation time | 4 | 2.831 | 0.708 | 2036.000 | < 0.001 |
| Exposure time | 6 | 1.276 | 0.213 | 611.664 | < 0.001 |
| Acclimation time × Exposure time | 24 | 2.364 | 0.099 | 283.408 | < 0.001 |
| Error | 70 | 0.024 | 0.000 |  |  |
| Total | 105 | 7.731 |  |  |  |
